# Frailty: the perioperative and anesthesia challenges of an emerging pandemic

**DOI:** 10.1007/s00540-023-03206-3

**Published:** 2023-06-13

**Authors:** Zhaosheng Jin, Joshua Rismany, Christopher Gidicsin, Sergio D. Bergese

**Affiliations:** 1 Department of Anesthesiology, Stony Brook University Health Science Center, Level 4, Room 060, Stony Brook, NY 11794-8480 USA; 2 Department of Neurosurgery, Stony Brook University Health Science Center, Stony Brook, NY 11794-8480 USA

**Keywords:** Anesthesia, Enhanced recovery, Frailty, Perioperative outcomes

## Abstract

Frailty is a complex and multisystem biological process characterized by reductions in physiological reserve. It is an increasingly common phenomena in the surgical population, and significantly impacts postoperative recovery. In this review, we will discuss the pathophysiology of frailty, as well as preoperative, intraoperative, and postoperative considerations for frailty care. We will also discuss the different models of postoperative care, including enhanced recovery pathways, as well as elective critical care admission. With discoveries of new effective interventions, and advances in healthcare information technology, optimized pathways could be developed to provide the best care possible that meets the challenges of perioperative frailty.

## Introduction

Frailty is a clinical syndrome characterized by reductions in physiological reserves across multiple organ systems. It is estimated that 15% of the over-65 population in the US fulfill the criteria for frailty, while 45% fulfill parts of the criteria for frailty. [1] Frailty has significant implications in the perioperative period, including prolonged postoperative recovery, increased risk for postoperative complications such as stroke, and increased risk of patients requiring discharge to a skilled care facility. With the aging population and increasing number of older patients undergoing surgery, perioperative management of patients with frailty will likely become one of the biggest challenges for healthcare providers. In this review, we will discuss the effect of frailty on postpositive outcomes, as well as some of the perioperative considerations.

## Definition of frailty

Frailty is defined as global reductions in physiological reserve across multiple organ systems, to the extent that the affected systems may approach clinical failure [2]. While old age is one of the main precipitants, multiple factors such as comorbidity, physical, cognitive and socioeconomic factors also contribute towards the development and progression of frailty. Younger patients can be considered frail by social aspects, cognitive aspects, and due to chronic diseases [1, 3].

In clinical practice, two schools of thought regarding the assessment of frailty are the acquired deficit and the frailty syndrome model. The “acquired deficit” model was first proposed by Rockwood et al.,[4] and is a quantitative analysis of the number of patient deficits (which includes comorbidities and functional impairments) according to a 70-item list. In this approach, frailty is the sum of the deficits. “Frailty syndrome” on the other hand focuses on specific features of frailty, which form its five diagnostic criteria (unintentional weight loss, weakness, slow walking speed, low physical activity, and self-reported exhaustion). In this approach, frailty is seen as a clinical syndrome distinct from functional impairment or disability alone. The two approaches, as well as the possible pathophysiology of frailty will be discussed in the next section [5].

## Pathophysiology

The explicit pathways that lead to frailty are not known, however, many theories exist. The current leading hypothesis of frailty is a proinflammatory state that occurs with aging also referred to as “inflammaging” [6, 7]. Proinflammatory markers cause changes at the cellular level leading to progressive cellular damage [8]. This causes a catabolic effect on muscle, impairment of normal homeostasis, and increased risk of morbidity and mortality from disease and environmental challenges [6, 8]. Changes in immune cell levels are believed to be responsible for the proinflammatory state; these include decreased naïve CD8 + T cells, CD4 + T cells, and CD19 + B cells, as well as inhibited T cells, and increased CD8 + T cells. Serum cytokines involved in “inflammaging” include IL-10, tumor necrosis factor (TNF- α), and micoRNAs. They are involved in gene regulation and modulation of cellular pathways including nuclear factor‐κB (NF-κB), mammalian target of rapamycin (mTOR), sirtuins, transforming growth factor‐β (TGF- β), and Wnt [9].

Wnt is a group of proteins that plays a role in development, aging, and carcinogenesis. In an animal study, Wnt pathway activation resulted in increased muscle fibrosis, while inhibition of the pathway led to increased myogenesis [10]. Similarly, Wnt pathway activation in mature kidney tissue has also been shown to increase renal fibrosis, while Wnt antagonists alleviate angiotensin induced podocyte dysfunction [11, 12].

Sirtuins are another set of important enzymes that are highly expressed in skeletal muscle, as well as the brain, heart, liver, and thymus, and have histone deacetylase or mono-ribosyltransferase activity. They protect DNA from both age-related and oxidative damage [13]. A decreased expression of sirtuins can lead to increased vulnerability to DNA damage, especially in the organs where they are highly expressed [13].

Activation of NF-κB, through muscle-specific transgenic expression of activated IκB kinase beta causes profound muscle wasting. Muscle wasting is seen in many pathologies including sarcopenia, cachexia, diabetes, immobilization, and denervation [14]. Decreased levels of anti-inflammatory cytokines, specifically IL-10 has been shown to replicate frailty in mouse models [15]. Frailty is also strongly associated with homeostasis disruption in energy metabolism and muscle activity [16]. This often manifests as sarcopenia in many frail patients [16].

Insulin resistance also seems to play a role in frailty through reduced regenerative ability, poor perfusion, oxidative stress, mitochondrial dysfunction, and inflammation [17]. Glucose is primarily taken up by skeletal muscle, and during hyperglycemia, all non-insulin mediated glucose uptake and 75–95% of insulin mediated glucose uptake occurs in skeletal muscle [18]. Muscle is also the main tissue for protein metabolism, and plays a critical role in prevention of acute illness and chronic disease [19]. Insulin-mediated growth of muscle mass is caused by activation of p38 MAPK and mTOR/p70S6 kinase, leading to stimulation of mRNA translation. This is impaired in insulin-resistance aged muscle. The result is poor nutrient utilization leading to a catabolic state and often resulting in sarcopenia [20]. This goes along with the idea that co-morbidities such as diabetes chronic kidney disease, chronic obstructive pulmonary disease, chronic heart failure, human immunodeficiency virus infection, and rheumatoid arthritis work synergistically with aging to cause frailty [21].

## Cognitive frailty

**Fig. 1:**
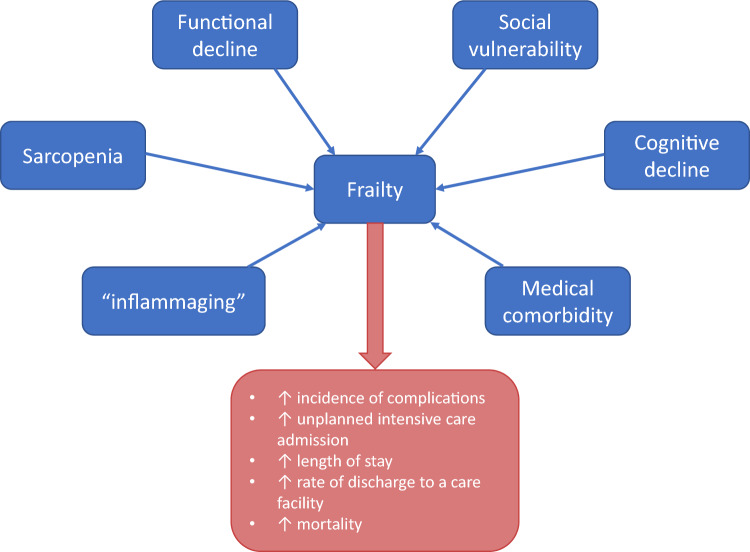
Comparisons between different domains of frailty

Definitions and assessments for cognitive frailty are heavily researched and debated. A definition for cognitive frailty is a state of reduced cognitive reserve caused by physical ailments other than neurodegenerative disease such as Alzheimer’s disease that is potentially reversible. The above definition has been introduced in 2013 by the International Academy of Nutrition and Aging and the International Association of Gerontology. In their consensus statement, cognitive frailty is defined as the simultaneous presence of physical frailty (frailty phenotype) and cognitive impairment (clinical dementia rating = 0.5) in older individuals without a definite diagnosis of dementia independent of other frailty dimensions (Fig. 1). De Roeck et al. suggest that cognitive frailty is distinct and can be seen without physical frailty in their Comprehensive Frailty Assessment Instrument-Plus where they looked at 5 domains of frailty; physical, psychological, social, environmental and cognitive [22]. To answer link between dementia and cognitive frailty, four studies in a small meta-analysis showed that patients with cognitive frailty had an increased risk of developing dementia as compared with those without. The prefrailty and cognitive impairment model demonstrated pooled HR = 3.99 [95% CI 2.94–5.43] and the frailty and cognitive impairment model demonstrated a pooled HR = 5.58 [95% CI 3.17–9.85] [23].

## Social frailty

A review article by Hobbelen et al. defines social frailty as a continuum of being at risk of losing, or having lost, resources important for fulfilling one or more basic social needs during the life span [24]. With less than 600 published articles and abstracts as of December 2022, social frailty remains an unexplored concept of frailty. Social frailty is associated with older age, being female, higher levels of education, lower income, unhealthy lifestyle and dissatisfaction with living environment (Fig. 1) [25]. Higher levels of education were the one of the most surprising associations with frailty. Fulfillment of basic social needs is necessary to function adequately and experience social wellbeing, just as basic physical needs fulfillment is required to experience physical wellbeing [24]. There are many theories on what comprises social frailty and social needs. One theory called social production functions specifies three distinctive social needs, the needs for affection, behavioral confirmation, and status. A considerable number contained factors relating to social frailty could be interpreted as social need fulfillment and social resources. Nearly all studies mentioned factors that could be interpreted as general resources in relationship to social frailty. Factors relating to social behaviors and activities, as well as self-management abilities, were mentioned rarely [24]. The results of a scoping review indicate that the threat of losing social and/or general resources should be a component of the concept of social frailty. Also, the absence social activities with friends and the threat of losing self-management skills and the ability to make decisions [24].

There seems to be a trend of physical frailty leading to social frailty in terms of decrease in social activities leading to cognitive decline and cognitive frailty [26]. This can often be seen in the elderly population and populations with decreased mobility leading to a decrease in social engagements and social frailty. Some studies also see the synergistic relationship between cognitive and social frailty [27, 28]. This relationship is seen in a study looking at gait speed and weakness showing a correlation to increased social frailty [28]. Interventions into improving mobility could be key to decreasing the risk of social frailty. Lee et al. showed that those classified as socially frail at baseline had an increased risk of developing physical frailty, compared with not socially frail participants (OR = 3.93, 95% CI 1.02–15.15) [29]. Surprisingly, a separate study showed no improvement in social frailty at 6 months following total hip and knee arthroplasty in patients with end-stage osteoarthritis despite improvement of physical frailty in most patients [30]. However, further investigation is warranted to assess the long-term outcomes of hip and knee arthroplasty on social frailty. While the underlying mechanisms of social frailty are not well understood, some theories exist. The recent COVID-19 pandemic and lockdowns worsened social frailty for many as there was decreased ability for neighbors, friends, and family to support each other. Another study believed that perceived loneliness; social media distress; social inequalities; loss of social and general resources; and physical and psychological stress has led to an increase in social frailty [31]. Studies have shown that perceived loneliness can lead to cardiovascular disease and mortality. Total peripheral resistance has a direct relationship with loneliness. Candidate mechanisms include age-related changes in vascular physiology, including increased arterial stiffness, diminished endothelial cell release of nitric oxide, enhanced vascular responsivity to endothelial constriction factors, increases in circulating catecholamines, and attenuated vasodilator responses to circulating epinephrine due to decreased beta-adrenergic sensitivity in vascular smooth muscle [32]. Loneliness can also lead to changes in the hypothalamic-pituitary adrenocortical axis and changes in immunoregulation including lower natural killer cell activities and higher antibody titers to the Epstein–Barr Virus and human herpes viruses [32]. Loneliness was associated to a greater brain amyloid-β protein burden that is seen in Alzheimer’s disease [31]. Social frailty independent of physical frailty has been shown to predict poor health outcomes including mortality.

## Frailty and perioperative outcomes

### Postoperative recovery

**Fig. 2:**
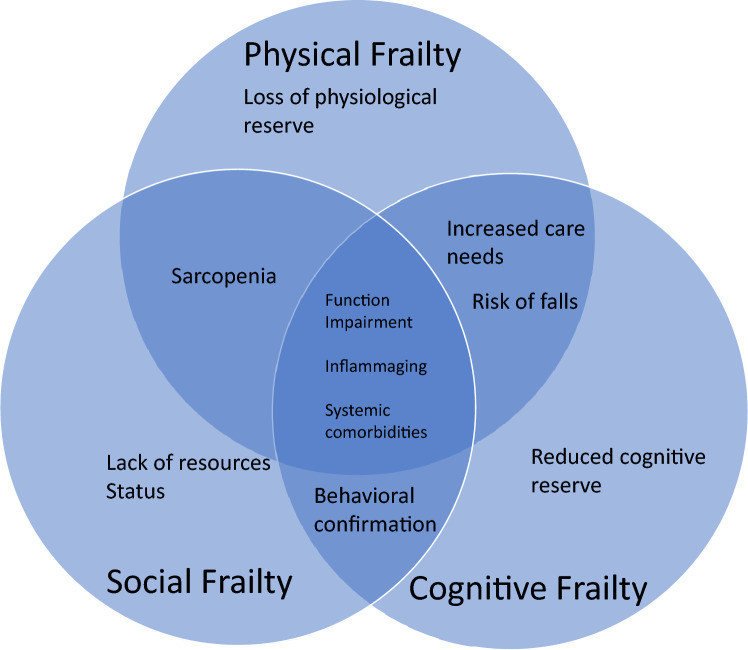
Schema of the contributory factors and perioperative outcomes of frailty

Frailty is often associated with a protracted course of postoperative recovery, with unplanned ICU admission, prolonged hospital stay, and discharge to skilled care facilities (Fig. 2). A study by Robinson et al. reported that after elective general surgery, almost 60% of patients with frailty required care facility on discharge [33]. Frailty has also been shown as an independent predictor for the development of postoperative disabilities and length of stay [34]. McIsaac conducted a cohort study of over 70,000 patients undergoing emergency general surgery, and reported that patients with frailty were twice as likely to require ICU admission, the length of stay in hospital was on average 10 days longer, and almost 30% of the patients with frailty required care facility on discharge, compared to 5% of patients without [35].

Using patient reported postoperative outcomes, McIssac et al. [36] examined the postoperative trajectories in patients with and without frailty who underwent elective, mostly orthopedic surgeries. The authors reported that while patients with frailty had higher disability score at baseline, this was most pronounced in mobility and life activities. One-third of the patients with frailty experienced early postoperative deterioration in their functional status but only 4.1% had persistently elevated disability score at 12 months. This pattern was not observed in patients without frailty. The authors suggested that patients with frailty could achieve meaningful functional recovery, which need to be balanced with the initial perioperative risk.

### Risk of complications

Frailty is also associated with significantly higher risk of postoperative complications. In a study of over 400,000 patients, Rothenberg et al. reported that frailty is associated with double the risk of postoperative complications after both ambulatory and inpatient surgeries [37]. Notably, frailty is associated with a threefold increase in the risk of pneumonia and pulmonary embolism, fivefold increase in the risk of CVA, and almost tenfold increase in the risk of AKI and myocardial infarction. They also reported that frailty is an independent risk factor, with a relative risk of 1.8–2.1, for unplanned hospital admission after surgery. Shinall et al. [38] demonstrated in a cohort study of over 400,000 patients and reported that patients with high frailty scores had clinically significant risk of mortality (more than 1%), even after low to intermediate stress surgeries such as arthroscopy and inguinal hernia repair. As discussed, frailty is multifactorial. Certain factors of frailty have been shown to increase different postoperative risks. In patients with reduced cognition preoperatively, there is a further decline in cognition postoperatively. Loneliness has also shown to lead to worse medical outcomes, including slower gait, increased mortality, and increased rate of cardiovascular insults after surgery. Recovery after surgery is more successful with a stronger support system. When patients lack a strong support, there is an increased risk of falling, malnutrition, and decreased motivation to recover [3].

Shah et al. conducted a cohort study of over 900,000 patients, and again found that frailty is associated with significantly increased risk of postoperative complication, as well as a higher risk of 30-day mortality. Interestingly, frail patients who have none or one (often minor) complications have disproportionately higher risk of mortality, this suggest that frail patients are at risk of decompensation and death after a relatively minor insult, likely due to the lack of physiological reserve [39]. This pattern was also seen in another study by Shinall et al.,[38] which reported that patients with higher frailty scores and experiences a single postoperative complication had significantly higher risk of mortality, this was not the case in patients with lower frailty score. Lin et al.[40] conducted a systematic review of 23 studies, and found that the studies consistently reported higher risk of complications, prolonged hospital stays, as well as short- and long-term mortality in patients with frailty.

It has been proposed that higher measured cumulative deficit is associated with worse postoperative outcomes. In non-surgical populations, patients with higher cumulative deficit index had higher risk of cardiovascular events and all-cause mortality [41]. In surgical patients, higher frailty is associated with longer-term outcomes, but not in-hospital or 30-day outcomes [42]. Han et al. [43] conducted a meta-analysis of studies which correlated frailty phenotype with postoperative outcomes and reported that patients with frailty phenotype had significantly higher risk of postoperative mortality and complications. In a head to head comparative study of Hopkins Frailty Score (a phenotype model) and the Modified Frailty Index score (a measure of cumulative deficit), 1042 prospective non-cardiac surgery patients were evaluated according to the two approaches preoperatively [44]. The author reported that both approaches had similar, but inaccurate predictive abilities in identifying patients at risk of prolonged hospital stay, postoperative complications and readmission.

Patients who are cognitively frail have worse clinical outcomes including disability, hospitalization, incident dementia, and death. Recent research shows the need for broad assessment to detect possible causes of cognitive frailty. This topic has caught attention due to the possible reversibility of cognitive frailty and the potential to prevent worse outcomes if patients are identified and interventions are set in place. Unfortunately, the evidence on the mechanism of cognitive frailty is not well understood and needs to be further studied. Research suggests that cognitive frailty can be caused by chronic inflammation, impaired hypothalamic–pituitary axis stress responses, energy, homeostasis dysfunction, endocrine dysregulation, mitochondrial dysfunction, oxidative stress, genomic factors, nutritional factors, metabolomic factors, gut dysbiosis, vascular risk factors and psychosocial factors. There is some evidence showing that the underlying mechanism behind physical and cognitive frailty are related furthering a strong link between frailty and cognitive impairment or dementia [26, 45]. Cognitive frailty has a low prevalence in the general population (1–1.8%) but, is seen quite frequently in clinical settings in 10–39% of patients. Cognitive tests such as the Mini Mental State Examination (MMSE) and the Montreal Cognitive Assessment (MoCA) are used to assess cognition. Cognitive frailty had a higher risk of all-cause mortality with a hazard ratio = 1.93% [95% CI 1.67—2.23] and dementia hazard ratio = 3.66% [95% CI 2.86—4.70)] compared with robust adults [46].

### Health care costs

Frailty is an independent risk factor for increased healthcare cost; this may not be surprising as a result of the increased care needs, management for postoperative complications, and longer hospital stay these patients often need. When controlled for the severity of complications, higher frailty score increases the health care cost by $20,000 to $80,000 [47]. In a study by McIsaac, frail patients who had elective surgical procedures, had a $30,000 higher healthcare cost [35]. Similarly, Robinson et al.[33] compared the in-hospital and post-discharge costs of patients undergoing colorectal surgery. The overall healthcare cost for patients with frailty was $77,000 higher than patients without; this included $49,000 in-hospital cost and $28,000 post-discharge costs. Another study by Eamer [48] also found that frailty was associated with significantly higher healthcare cost after discharge.

### Preoperative assessment and optimization

Preoperative recognition of frailty is vital for perioperative care planning. During the preoperative assessment, frailty assessment should be completed in elderly patients. Several frailty assessment tools have been developed for clinical use. Edmonton frailty scale is a 11-item assessment tool with variable weighting and a total score of 17, the assessment could be completed with a short interview and tasks; those scoring more than 5 are described as various stages of frailty [49]. Another assessment tool is the clinical frailty scale, which is a 9-point linear scale from “very fit” to “terminally ill,” both the medical history and functional capacity of the patient is taken into consideration [50]. A recent study compared clinical frailty scale to the modified Fried scale (a 5-category assessment consist of interview and tasks), the authors reported that there were no significant differences in the detection of postoperative mortality and disability, but the preoperative physicians noted that the clinical frailty scale was significantly easier to use [34].

One limitation of the frailty assessment tools is that while it is useful for identifying patients with frailty, they are not sufficiently detailed for identifying the care needs of the individuals. Comprehensive geriatric assessment is a systematic, multidisciplinary approach for assessing older patients with complex care needs. The components of a comprehensive geriatric assessment includes expert led medical review, as well as social, functional and psychological review, followed by patient centered perioperative care planning [51, 52]. Partridge et al. [53] conducted a systematic review, which reported that preoperative comprehensive geriatric assessment is associated with significantly lower cancellation rate, shorter hospital stay, as well as lower risk of postoperative complications.

In the preoperative period, patients should have their medications reviewed, and they should be counseled on which medications to continue and which medications to hold in the perioperative period. Any medications that are not required should be discontinued to help avoid complications with polypharmacy. In addition, medications with potential for withdrawal, or with interactions with anesthetics should be identified [54].

It is recognized that frail patients are at significant risk of perioperative functional decline, which will negatively affect postoperative recovery. One approach has therefore been to pre-emptively optimize the functional reserve prior to surgery through exercise and nutrition, termed “prehabilitation”. Mina et al. [55] conducted a multi-center study which randomized cystectomy patients to 3–4 h of moderate exercise or usual care, and followed patients for up to 26 weeks after surgery. The authors reported that the prehabilitation cohort had significantly lower preoperative anxiety and body fat percentage; postoperatively, the prehab cohort also had significantly longer 6 min’ walk distance and higher grip strength. A systematic review and meta-analysis of 22 studies found that the prehabilitation cohort had significantly shorter length of stay; the authors also reported that qualitatively, most studies reported that prehabilitation was associated with significant improvement in patients’ musculoskeletal task performance [56]. Another meta-analysis on patients who underwent abdominal surgeries found that prehabilitation significantly reduced the risk of postoperative complications, but not the length of hospital stay; qualitative analysis of functional capacity suggested that prehabilitation may improve respiratory muscle strength, but not the overall fitness level [57].

Despite the promising results regarding prehabilitation, there are two areas of uncertainty regarding its implementation. Firstly, prehabilitation is a resource intensive process that may require significant changes to preoperative pathways; the quality of evidence with regards to prehabilitation is also notably weak due to various study limitations. It is not clear if the implementation of prehabilitation is likely to be cost-effective. Secondly, there is currently limited evidence on prehabilitation of patients with frailty. While some studies have shown that exercise programs in patients with frailty may improve physical performance [58, 59], it is not clear if this will translate into improved perioperative outcomes [60]. On the other hand, it is possible with further research and advances in optimization programs, prehabilitation may impart a significant benefit to patients with frailty.

## Intraoperative considerations

### Choice of anesthesia

Anesthesia and surgical stress are associated with a number of postoperative complications, including atelectasis, pneumonia, myocardial injury, acute kidney injury and postoperative delirium [61], and the risk is significantly higher in patients with frailty [37]. In patients with frailty or high risk of complications, the use of regional anesthesia may help mitigate some of the perioperative risks such as pulmonary complications and postoperative delirium [62–64]. On the other hand, it is not clear if regional anesthesia improves postoperative survival in patients with frailty [65, 66]. Peripheral nerve blocks often have residual analgesic effect up to 24 h after surgery, this would reduce postoperative opioid requirement and may further reduce the risk of respiratory depression and falls [67, 68]. The benefit of techniques such as neuraxial anesthesia, transverse abdominis plane (TAP) block, brachial plexus block and lower limb nerve blocks have been demonstrated consistently in a number of meta-analyses [69–73]. The efficacy of regional anesthesia could also be further augmented through the use of adjunct medications, such as dexmedetomidine[74] and dexamethasone [75].

### Pharmacological consideration during general anesthesia

When providing general anesthesia for patients with frailty, expert opinions advocate the use of depth of anesthesia monitoring modalities such as the bispectral index (BIS) [76, 77]. Clinical trials and meta-analyses have shown that depth of anesthesia monitoring can reduce the risk of excessive anesthetic agent administration.[78] This is associated with significantly faster emergence from anesthesia [78, 79], and may also reduce the risk of postoperative delirium[80] and intraoperative hypotension [81]. However, there is limited direct evidence that depth of anesthesia in patients with frailty improves postoperative outcomes.

It is thought that 50–60% of patients may have residual NMJ blockade after emergence from anesthesia [82], this significantly increase the risk of postoperative pulmonary complications [83]. The use of quantitative NMJ monitoring, a short acting NMJ blocker, and appropriate reversal at the end of surgery are vital for minimizing such risks [84].

While intraoperative opioids are common component of balanced anesthesia, it should be used judiciously and titrated with caution in patients with frailty its use in patients with frailty factors, such as increased opioid sensitivity, altered drug distribution and metabolism [85].

Dexmedetomidine is a highly selective α-2 adrenergic agonist with sedative, analgesic, and neuroprotective effects. Intraoperative administration of dexmedetomidine reduces perioperative opioid requirement [86, 87]. In addition, perioperative administration of dexmedetomidine has also been shown to reduce the risk of postoperative delirium [88, 89].

Lidocaine is an amide local anesthetic which inactivates voltage gated sodium channels, but also has anti-inflammatory and neuroprotective effects [90]. More recent studies have showed systemic administration of lidocaine at doses 1.5 to 3 mg/kg/hour in the perioperative period is safe [91]. Vigneault et al. conducted a meta-analysis of 29 studies, and reported that perioperative lidocaine infusion (with or without initial bolus) is associated with significantly lower postoperative pain score, opioid requirement, and opioid related adverse events such as PONV and constipation [92].

Antibiotics should be given to frail patients if indicated perioperatively [93]. Antibiotics that are tolerated well and have lower rates of interaction with other medications should be preferred in this population due to poly-pharmacy [93]. First-generation and second-generation cephalosporin are preferred for this reason [93]. There should be consideration given to the patients’ antibiotic resistance due to previous bacterial infections [93].

As with the management of frailty in most medical context, the focus is personalized, and judicious use of medications based on a patient’s medical history. For minor surgical procedures, there may be merit to the ‘less is more’ approach; while in major surgeries, the benefit of preemptive interventions must be balanced with the risk of polypharmacy and drug adverse events.

### Intraoperative homeostatic considerations

It is thought that preoperative fasting, intraoperative insensible losses, and vasomotor depressive effects of anesthesia all predispose patients to hypovolemia; as a result, intravenous fluid administration is common practice during surgery. However, it is increasingly recognized that excessive fluid administration is also associated with complications such as bowel edema and respiratory failure [94]. This is particularly important in patients with reduced physiological reserve, who are at increased risk of decompensation from fluid overload. Goal directed fluid therapy describes a clinical approach of administering intravenous fluids according to pre-defined hemodynamic targets [95], with the aim of optimizing preload and intravascular volume. Hemodynamic parameters may be obtained from a variety of invasive (arterial catheter, central venous catheter) and non-invasive (pulse plethysmography) means, and are used to predict patient’s fluid responsiveness. This allows for targeted administration of IV fluids (and vasopressors) to optimize cardiac output and perfusion.

A meta-analysis of 45 randomized controlled trials found that the use of goal directed fluid therapy is associated with significantly lower risk of morbidity and mortality, as well as faster recovery of bowel function [96]. Makaryus et al.[97] proposed a risk stratified approach which accounts for patient and procedure related risks. Considering the perioperative risks associated with frailty, such patients may benefit from intraoperative hemodynamic monitoring (such as arterial catheter or pulse plethysmography). In frail patients undergoing high-risk surgeries, invasive monitoring such as transesophageal doppler may be considered to accurately monitor patient’s fluid status and to predict the most effective intervention should the patient develops hypotension or hypo-perfusion.

Frailty causes significant changes in the homeostasis of patients intraoperatively and is at increased risk due to dysfunction and decreased preserve of most organ systems. A retrospective study showed frail patients had less intraoperative variability in mean arterial pressure which was associated with increased 30-day post-operative mortality [98]. Autonomic dysregulation is one of the causes of increased perioperative complications in the frail population [98]. One factor that plays a role in prolonged hypotension is diastolic heart failure and decreased venous compliance that is common in the elderly [99]. Due to the pulmonary changes in the frail population there is increased alveolar-arterial gradient, susceptibility to hypercarbia and hypoxemia, susceptibility to residual anesthetic effects, increased work of breathing, and increased dead space ventilation [54]. Impaired glucose tolerance can lead to intraoperative hyperglycemia [54]. The decrease in muscle mass and autonomic dysfunction increases the risk of hypothermia [54].

### Temperature monitoring

Interoperative temperature management is of vital importance, especially during general anesthesia cases lasting greater than 30 min. Elevated temperatures can be seen in a variety of pathologies including malignant hyperthermia, infectious fevers, blood in the fourth ventricle, and blood transfusion reactions. Hypothermia is more common because many anesthetics cause vasodilation and impaired thermoregulation. Hypothermia can lead to poor wound healing, increased wound infections, coagulopathy, increased blood transfusions, and delayed recovery from anesthesia [2, 100].

Vigilant temperature monitoring allows for faster adjustments to warming measures and faster diagnosis and treatment of hyperthermic pathologies. Compared to passive warming, active warming is more effective and is necessary to maintain normothermia when general anesthesia is given for greater than one hour. Complications due to hypothermia can be increased in the frail population making temperature monitoring even more crucial in this patient population [3, 101].

## Postoperative care

### Analgesia

Postoperative pain is common after major surgery, with up to 30% of patients experiencing severe pain after surgery, this can lead to prolonged hospital stay, as well as higher risk of unplanned re-admission [102]. Postoperative pain impairs postoperative functions, such as ambulation, oral intake and sleep. It can also induce excessive activation of stress and sympathetic responses. Postoperative pain has been shown to increases the risk of atelectasis and subsequent pulmonary complications, it is also associated with increase the risk of postoperative delirium [103], and may contribute to the development of postoperative cardiac complications [104]. On the other hand, perioperative opioid use is also associated with significant risk, this includes constipation, post-operative nausea and vomiting (PONV), sedation and respiratory depression [105–107]. Postoperative opioid related adverse events are associated with longer length of hospital stay and risk of mortality.[106, 108] The current best practice is therefore to administer multimodal, opioid-sparing analgesia after surgery.

Acetaminophen is a centrally acting anti-pyretic and analgesic, it is widely used in the perioperative setting, often in a ‘round the clock’ regimen to maximize its analgesic efficacy. While it is generally thought to be safe, it has been suggested that chronic use may be associated with renal injury [109]. NSAIDs are also effective for postoperative analgesia and opioid minimization [110], but their use are limited by concerns of AKI as well as cardiovascular risks. Interestingly, two recent cohort studies have reported that in patients undergoing major surgery, perioperative NSAIDs did not increase the risk of AKI [111, 112].

Ketamine is an NMDA receptor antagonist, with additional action on the mu opioid receptor, GABA receptor, and muscarinic acetylcholine receptor [113]. Perioperative ketamine is effective in reducing opioid requirement and PONV [114]. While an anesthetic dose of ketamine is associated with the risk of an emergence reaction, a meta-analysis has reported that analgesic doses of ketamine do not increase the risk of postoperative delirium [115]. However, ketamine is contraindicated in patients with ischemic heart disease and heart failure due to its chronotropic and negative ionotropic effects [116].

Gabapentinoids are thought to down-regulate excitatory neurotransmitter release by blocking voltage-gated calcium channels and NMDA receptors [117]. Two cohort studies of patients undergoing laparoscopic surgeries and joint arthroplasty reported that preoperative administration of pregabalin and gabapentin was associated with significantly higher risk of respiratory depression [118, 119]. This led to the release of a Food and Drug Administration (FDA) drug safety communication warning against the use of gabapentinoids, particularly in conjunction with other central nervous system depressants such as opioids [120].

Non-pharmacological interventions for postoperative pain includes cryotherapy, relaxation therapies, and transcutaneous electrical nerve stimulation (TENS) [121–123]. These are effective adjunct therapies in selected patients and procedures, and are associated with very few systemic side effects, but have limited efficacy in moderate to severe pain.

Patients with frailty often have limited physiological reserve in tolerating postoperative pain response and adverse drug effects. There is likely no ‘on-size-fits-all’ multimodal analgesia that would be safe and effective in all patients with frailty. However, judicious use of all medications with respect to the patients’ medical considerations, and optimized implementation of non-pharmacological interventions will likely lead to the best outcome.

### PONV

Postoperative nausea and vomiting (PONV) is a common adverse event after surgery and anesthesia, estimated to affect 30% of the surgical population. In addition to being distressing to the patient and leading to prolonged postoperative anesthesia care unit (PACU) stay, PONV can also impair oral intake and postoperative recovery. Gan et al. published an updated iteration of the PONV consensus guideline, and now advocate for the use of general multimodal prophylaxis, using two or more prophylactic interventions as a standard of care [124].

There is very little evidence regarding the management of PONV in patients with frailty, but several classes of antiemetics are associated with increased risk of adverse effects in the older population, and may be contraindicated. This includes drugs with anti-cholinergic effects, which can precipitate postoperative delirium, such as scopolamine, promethazine and prochlorperazine; the use of dexamethasone is also cautioned due to the risk of postoperative delirium.

Serotonin antagonists such as ondansetron are widely used as first line PONV prophylaxis. Serotonin abnormality is thought to contribute to the development of postoperative delirium, and has been hypothesized that serotonin antagonists may in fact be protective against postoperative delirium. Hauqe et al. [125] conducted a systematic review, which reported that postoperative ondansetron is associated lower risk of delirium compared to haloperidol or placebo. Aprepitant is a neurokinin antagonist which has demonstrated good antiemetic efficacy. It is administered *per os,* but fosaprepitant is available as an intravenous formulation. Side effects of aprepitant include constipation and hypotension. Based on studies from both PONV and chemotherapy related nausea and vomiting, the Food and Drug Administration (FDA) have approved the use of aprepitant in the older population without dose adjustment [113]. In institutions which have access to aprepitant, it could be a viable second line PONV prophylaxis and treatment in the older population [126].

### Medications

The American Society of Anesthesiologists (ASA), has no specific guidelines for medications for frail patients other than to follow beers criteria of medications to avoid in the elderly (Table 1) [127]. However, pathophysiologic changes including increased adipose tissue and decreased total body water and decreased muscle mass have effects on the pharmacokinetics of the medications given. Medications with higher lipophilicity will have larger volumes of distribution and can have longer duration of actions [99]. Hydrophilic medications will have higher peak plasma concentrations [99]. Renal mass and speed of renal excretion of medications are also decreased, and due to the decreased muscle mass, creatinine may not reflect the worsening renal function [99]. Medications with renal toxicity should be avoided, if possible [99]. Hepatic metabolism of medications are slower due to reduced hepatic blood and decreased activity of the cytochrome P450 system thus affecting phase 1 metabolism of medications [99]. Poor airway reflexes can lead to residual weakness after many medications with special consideration to medications that cause paralysis or sedation [99]. Overall, the pathophysiology of a frail patient must be considered when choosing medications and must be tailored for each patient. An international task force in 2019 evaluated the evidence presently available for pharmacological treatment for the management of frailty. They concluded that they do not recommend pharmacological treatment for the management of frailty due insufficient evidence to support any pharmacological treatment [128]. The task force also did not recommend vitamin D supplementation unless vitamin D deficiency is present [128]. Patients should have their medications reviewed for poly-pharmacy and the minimum number of medications required should be used [54]. Medications used to treat delirium should be limited to only patients who pose harm to themselves or others [99]. Beers criteria should continue to be used as guide for medications to avoid in this population [127, 129]. As discussed previously, with intraoperative medications the pathophysiology of the frail patient must be considered when determining medications [99]. Postoperative medication management is perhaps one of the most challenging aspects in the management of frailty due to the complex interplay between baseline comorbidity, post-surgical changes and patient needs. Beer’s criteria provides a basic framework for the perioperative medical management, further provider expertise is highly valuable in this field, as is the involvement of geriatric specialist in higher risk patients.

**Table 1:** Example of common perioperative medications that should be avoided according to the Beers criteria[129]

Promethazine
Scopolamine
Metoclopramide^a^
Antipsychotics^b^
Atropine
Benzodiazepine
Meperidine
Methocarbamol
Non-steroidal anti-inflammatory drugs

^a^Except for gastroparesis treatment duration < 12 weeks

^b^Except for schizophrenia, bipolar disorder, short term use as an antiemetic during chemotherapy

### Nutrition

Enteral nutrition should be started as soon as possible after surgery especially for patients with gastrointestinal surgery [54]. Patients who cannot be fed orally should be considered for tube feeds within 24 h of surgery [54]. Recent systematic reviews indicate that in observational studies, under-nutrition in community-dwelling older adults is closely associated with frailty [130, 131]. However, when intervention trials are considered, evidence is heterogeneous and only very low certainty evidence supports protein/caloric supplementation for older adults with frailty, so no definitive recommendation has been made [128, 132]. Poor nutrition remains an issue in the frail population and getting this population to the ideal nutritional status is challenging, however, starting feeding early has been the strongest recommended advice.

### Mobility and falls

Frail patients are at increased risk of falling and should have an evaluation for fall risk. [54] Universal fall precautions should be highly considered in all frail patients, however, it should not delay early postoperative mobilization [54]. Early mobilization is strongly recommended by the American Geriatric Society, however, the evidence behind the recommendation is weak [133]. Targeted interventions should be in place for patients to prevent falls [54]. Patients with altered mental status should be assessed for delirium, have frequent checks, and undergo a medication review [54]. Patients should be monitored for dehydration and for orthostatic hypotension and be adequately hydrated [54]. Patients who have to use the bathroom frequently should have scheduled times to be assisted. Patients with history of falls should have a walking device at bedside if they use one at home, and have their room checked for hazards. In addition, special attention should be given to those patients on anticoagulation medications [54]. Patients with visual impairment should have their corrective eye glasses within reach [54]. A task force concluded that multicomponent physical activity should be recommended for all older adults with frailty [128]. However, in a randomized clinical trial there was no difference seen in postoperative outcomes when frail patients had prehabilitation compared with postoperative rehabilitation [134].

### Complication management

Adverse surgical outcomes associated with frailty include mortality, non-routine recovery, need for resuscitation, delirium, major adverse cardiac and cerebrovascular events, increased length of stay, discharge to facility, readmission, functional decline, and decreased quality of life [40]. Thirty-day mortality was 4 times higher in frail patients [40]. Cardiac rehabilitation post-operatively in patients with valvular heart disease may improve outcomes and decrease the severity of frailty [135]. Pain should be addressed using multimodal pain control and opioids should be avoided, if possible [133]. Postoperative delirium is common in the frail population and use of multidisciplinary teams, early mobility and walking, avoiding restraints, improving sleep hygiene, and adequate nutrition, fluids and oxygen are strongly recommended. Although evidence for these recommendations were low, benefits greatly outweighed risk [133, 136]. In the ICU setting, dexmedetomidine may be used reduce the risk of postoperative delirium in patients requiring postoperative mechanical ventilation [133]. Care teams should look for and address precipitating causes of delirium, including but not limited to presence of infection, hypoxia, electrolyte abnormalities, urinary retention, fecal impaction, pain, medications, hearing or vision issues, language barriers, or cognitive decline [54]. With increased risk of PONV, patients should be assessed for risk and receive prophylactic interventions to decrease nausea and vomiting [54]. To prevent pressure ulcers and nerve injury, attention should be paid to positioning of the patients so they have a softer surface on bony prominences and limit pressure on peripheral nerves [54]. Pulmonary complications can be best prevented using aspiration precautions, having patients use an incentive spirometer, use of deep breathing exercises, and consideration of epidural analgesia when appropriate [54]. A study showed high performance hospitals determined by the lowest mortality have the best survival rates consistently regardless of the surgery [137]. The cause of the difference in mortality was not definitively understood and further research is necessary to identify the causes [137]. Frail patients may benefit from having surgeries performed at the high performing institutions [137]. The data on the complications of frailty postoperatively is thoroughly discussed in the literature. The preoperative assessment is a very helpful prognostic indicator. However, the data on reducing complications postoperatively is limited and heterogenous, and is an area for further investigation with interventional studies.

### Expedited and emergency surgery

In patients with frailty who are undergoing expedited surgery, one of the common concerns is the time limitation in preoperative optimization. In patients undergoing cancer surgery, frailty is a significant predictor of postoperative morbidity and mortality, especially in patients who otherwise have low grade or early-stage cancers [138]. While prehabilitation has demonstrated success in the cancer surgery population, studies to date have indicated limited efficacy of prehabilitation of cancer surgery patients with frailty [134, 139, 140].

Similarly, frailty has been shown to increase the postoperative morbidity after emergency surgery [141, 142], this is independent of the surgical procedure risk. It is not clear if this is modifiable through optimized perioperative pathways.

### Alternatives to usual postoperative care

Studies in older trauma patients showed preserved activities of daily living at 1-year postoperatively and improved adherence to geriatric management guidelines for patients who received a geriatric consult compared to usual care [143, 144]. Furthermore, close involvement of the geriatric specialist in directing postoperative care has been shown to reduce postoperative complications, as well as the length of hospital stay [145, 146]. Alternatively, there are studies that report that routine admission of high-risk postoperative patients to critical care units, where they could be closely monitored for signs of postoperative decompensation could reduce the occurrence of overt complications and improve outcome [147–149]. There is a strong recommendation to offer social support to frail patients to help with the various obstacles frail patients face including isolation, falls, and nutrition [128].

### Enhanced recovery pathways for patients with frailty

Enhanced Recovery After Surgery (ERAS) pathways represent a multidisciplinary, evidence-based approach in optimizing postoperative recovery and reducing complication risks [150]. The adoption of ERAS pathways have significantly reduced the length of hospital stay, which is beneficial for patients as well as healthcare institutions. Several studies report that older and frail patients enrolled into enhanced recovery programs have significantly longer lengths of stay and higher rates of complications [151, 152]. This, however, does not take into account the naturally longer postoperative recovery in older or frail patients. Indeed, while some studies reported lower protocol compliance in patients with frailty [153], others have reported that frailty is not associated with significantly lower compliance with ERP interventions [154, 155]. Moreover, when compared to conventional postoperative management, enhanced recovery interventions in elderly and frail surgical patients are associated with significantly shorter length of stay, with no significant increase in complication or readmission rates [155, 156].

## Conclusions

With ever increasing number of older patients with functional limitations and medical comorbidities undergoing higher-risk surgical procedures, perioperative frailty will be a significant challenge faced by clinicians around the globe. Due to the multisystem changes associated with frailty, the changes in perioperative physiology and complication management can be complex. On the other hand, increasing clinician experience in managing perioperative frailty has led to the development of targeted interventions, as well as the implementation of multidisciplinary pathways, optimized to meet the care needs of such patients. With the advances in healthcare information technology, more complex intervention and outcome metrics could be assessed at a system level over time, to ensure that healthcare resources are allocated effectively and provide the best care possible to meet the challenges of perioperative frailty.
